# Corneal grafting: what eye care workers need to know

**Published:** 2009-12

**Authors:** David Yorston, Prashant Garg

**Affiliations:** Consultant Ophthalmologist, Gartnavel Hospital, 1053 Great Western Road, Glasgow G12 0YN, Scotland.; Associate Director, Cornea and anterior segment services, LV Prasad Eye Institute, Hyderabad, India.

**Figure:**
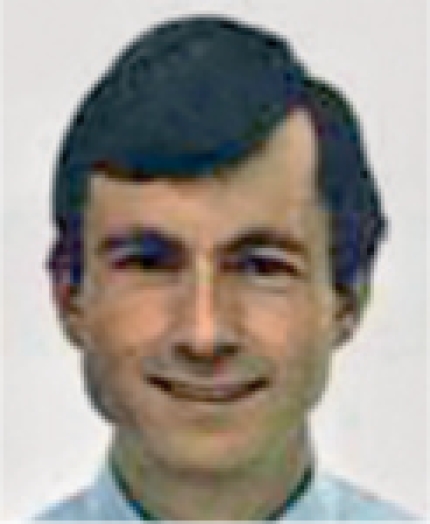


**Figure:**
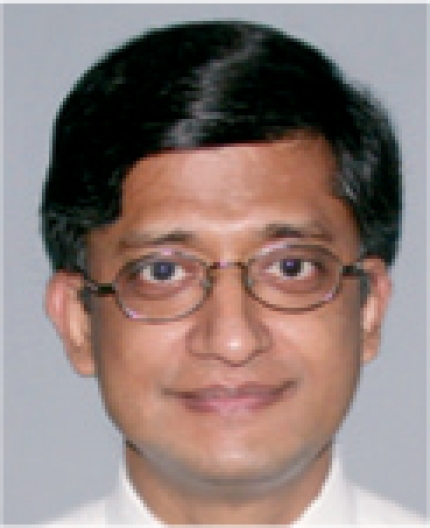


## Introduction

We know that many of the most common causes of corneal blindness: vitamin A deficiency, trachoma, and ophthalmia neonatorum, can be prevented by simple primary health care measures. Some conditions are more difficult to prevent, but can be effectively treated, such as suppurative keratitis (page 39) or herpes simplex keratitis. However, some patients will still develop blinding corneal disease. What can be done for them?

At present, the best hope for curing corneal blindness is a corneal transplant, also known as a corneal graft.

In contrast with most other transplants, corneal grafting is relatively straightforward. Although additional surgical training is needed, the operation itself requires little equipment beyond a standard cataract set and a good operating microscope. Because there are no blood vessels in the cornea, the likelihood of rejection is less than for other transplants.

In this article, we want to provide guidance to eye care workers who want to know who should be referred for a graft and what complications they may need to manage after patients have had their operation.

## What is corneal grafting?

In this operation, the central 7–8 mm of the patient's own diseased cornea is removed. A similar-sized disc of donor cornea is then inserted and sutured into position. In most cases, the full thickness of the cornea, including epithelium, stroma, and endothelium, is transplanted; this is known as a penetrating graft. However, it is also possible to transplant just the outer, anterior layers (stroma and epithelium) or the inner, posterior layers (endothelium and Descemet's membrane).

**Figure:**
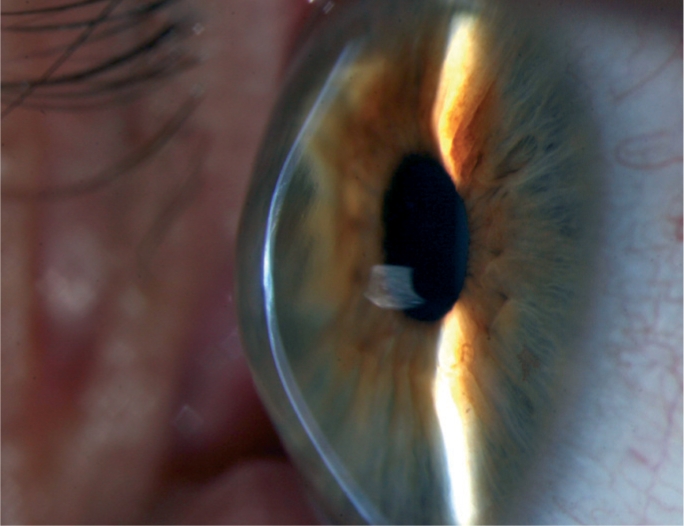
Figure 1. Keratoconus. Note the central scarring, with thinning and ectasia

David Yorston

## Indications and prognoses

Any corneal disorder that causes visual impairment may be an indication for a corneal graft. However, the prognosis for corneal grafting varies greatly.

The risk of rejection is higher if the cornea is vascularised (contains blood vessels), or is inflamed or perforated (as is liable to occur in severe suppurative keratitis, for example). The same holds true for any eye that has generalised disease of the ocular surface.

Table 1 lists some of the different diagnoses that may be treated by a corneal graft, along with their prognoses. Conditions with a "very poor" prognosis should not be treated by corneal grafting as this is a waste of valuable transplant tissue.

**Table 1:** Table 1. Indicaions for corneal graft and their prognoses

Diagnosis	Prognosis
Keratoconus	Excellent
Corneal dystrophies, such as lattice, granular, Fuch's	Excellent
Corneal scar - healed ulcer	Moderate
Bullous keratopathy - aphakic or pseudophakic	Moderate
Herpes simplex keratitis	Moderate
Corneal scar: active ulcer/keratitis, threatened perforation (grafting may be done to salvage the eye, rather than vision)	Poor
Corneal scar: trachoma	Very poor
Ocular surface disorder: chemical burn, Stevens-Johnson syndrome	Very poor
Mooren's ulcer	Very poor
Re-graft	Very poor

The visual prognosis for a corneal graft is also affected by the state of the rest of the eye, not just the cornea and ocular surface. Uncontrolled intraocular pressure (IOP) is a contraindication to grafting, as IOP control is likely to be worsened by a corneal graft. Patients with known posterior segment disease affecting the retina or optic nerve are also unlikely to benefit.

A corneal graft requires much more postoperative care than a cataract extraction. This entails repeated clinic visits and using expensive eye drops frequently; therefore, the patient must value their new cornea and be motivated to take care of it.

Patients are most likely to value their new cornea if the grafted eye is their better eye. In general, this means that patients with unilateral disease and perfect vision in the other eye are poor candidates. There are exceptions: for example, if the eye is painful as well as visually impaired.

Corneal grafting in children has a very poor prognosis and requires more intense postoperative care; it should therefore be considered carefully.

## Outcomes

Studies from South India^1^ showed that 69 per cent of grafted corneas were clear at two years after surgery. In East Africa,^2^ 87 per cent of grafts for keratoconus survived for at least two years, compared to 65 per cent performed for other diagnoses.

Unfortunately, a clear graft does not guarantee good vision. Patients may have coexisting problems, such as glaucoma, cataract, or amblyopia. Because corneal grafting alters the shape of the cornea, it often causes significant astigmatism, which can be difficult to correct with spectacles.

Visual outcomes in East Africa^2^ were much better for patients with keratoconus than for those with other diagnoses. In patients grafted for keratoconus, 33 per cent were <6/60 in both eyes preoperatively, compared to 5 per cent post-operatively; 78 per cent could see 6/18 or better following surgery. This data showed that corneal grafting is an effective cure for blindness caused by keratoconus.

This also demonstrates the importance of monitoring and reporting the outcomes of corneal graft operations.

## Complications

The main causes of graft failure in both South India^1^ and East Africa^2^ were graft rejection and infectious keratitis. Both of these complications are treatable and often preventable. In many cases, graft failure could have been prevented by early and effective management at a local eye clinic.

### Rejection

Graft rejection is caused by an immune reaction directed against the foreign endothelial cells of the transplanted cornea. Approximately 20–30 per cent of penetrating grafts will have a rejection episode at some time. The most common symptoms of graft rejection are blurred vision, light sensitivity, redness, and pain; patients should be advised to attend an eye clinic immediately if they develop any of these symptoms. Rejection is recognised by the appearance of corneal oedema in a previously clear graft. The oedema usually spreads upwards across the graft from the inferior edge of the transplanted cornea. The eye is often inflamed and a line of keratic precipitates may be seen at the edge of the oedematous cornea.

**Preventing rejection** begins with selection for surgery. Some diagnoses, such as keratoconus and other corneal dystrophies, have a very low risk of rejection. Following surgery, rejection is prevented by topical steroid drops. These are used at different frequencies for varying lengths of time, depending on the underlying diagnosis and the perceived risk of rejection. Whatever steroid regime is used, the drops should never be suddenly stopped, but should always be tailed off gradually. The usual duration of steroid therapy following a full thickness graft is six months in phakic patients and twelve months in pseudo-phakic or aphakic patients.

With prompt diagnosis and immediate treatment, graft rejection can often be reversed. The recommended **management of graft rejection** is intensive steroid treatment, initially in the form of hourly topical steroid drops. The use of systemic steroids, such as 500 mg methylprednisolone, has been shown to make little difference to the outcome and the authors therefore do not recommend it.

### Infection

The second common cause of graft failure is suppurative keratitis. This presents in the same way as any other microbial keratitis (page 39). Patients with corneal grafts are at increased risk of corneal infection because they have reduced corneal sensation and are often on long-term topical steroids. The management of infectious keratitis is the same in these patients as it is for anyone else. A scraping should be taken for gram stain and culture if available (see page 42). Intensive antibiotic treatment, either monotherapy with a topical fluoroquinolone (such as ofloxacin) or combination treatment with a cephalosporin (such as cefuroxime) and an aminoglycoside (such as gentamicin) is started immediately after the scraping has been taken, and is continued for at least 48 hours. In some settings, anti-fungal treatment may be required.

### Loose suture

The most common predisposing factor for infectious keratitis following a corneal graft is a loose suture. With time, as the graft wound heals, the very fine sutures either break or become loose. In both cases, they will erode through the corneal epithelium. This destroys the barrier effect of the epithelium and allows microorganisms to get into the cornea where they cause infection. A loose suture also promotes the growth of blood vessels into the cornea, which can lead to rejection.

All loose or broken sutures which protrude through the epithelium should be removed immediately. They can be detected by staining the cornea with fluorescein. Eye workers are sometimes reluctant to remove a corneal suture for fear that it may be the only thing holding the cornea together! This is an understandable reaction; however, if the stitch is loose or broken, then it cannot be providing any support to the wound. It is not serving any useful purpose and greatly increases the risk of complications, and it should be removed urgently.

To remove the suture, give local anaesthetic drops three times and wait three to five minutes. Cut the suture with the sharp edge of a 26G needle. Using a pair of very fine forceps, grasp the end of the stitch and pull it gently out of the eye. Always give antibiotic drops for five days afterwards to prevent infection.

**Figure:**
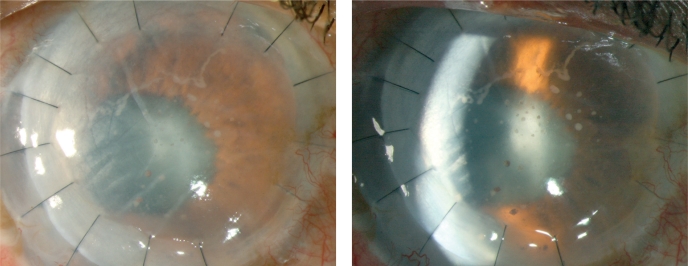
Figure 2. Graft rejection. Note oedema in the lower two-thirds of the graft (Figure 2a) along with multiple keratic precipitates (Figure 2b)

David Yorston, David Yorston

**Figure:**
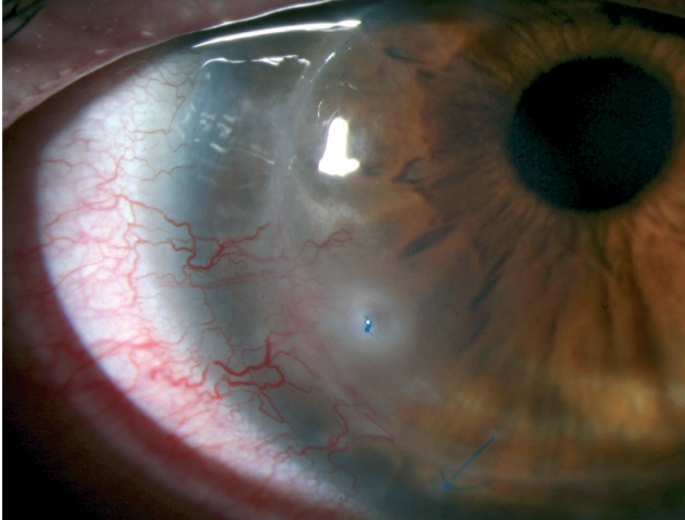
Figure 3. A broken stitch has caused blood vessels to grow into the corneal graft, increasing the risk of rejection. It is surrounded by infiltrate, which may indicate infection

David Yorston

## Summary

- Most corneal blindness can be prevented, but for those patients who have bilateral visual impairment caused by corneal disease, a corneal graft is the only hope of restoring sight.
- The best candidates for a corneal graft are patients with keratoconus or other corneal dystrophy, in whom about 90 per cent of grafts will remain clear for at least two years.
- Good postoperative care is essential. Patients must remain on topical steroids for a long time, and these should never be stopped suddenly.
- Graft rejection is often reversible if it is treated immediately with intensive topical steroids.
- All loose sutures should be removed immediately to reduce the risk of microbial keratitis.
